# Neocyte‐enriched blood does not provide a survival advantage for red cells when compared to conventional filtered blood

**DOI:** 10.1111/vox.70085

**Published:** 2025-07-27

**Authors:** Adriaan Meyer, Heather Hendrickse, Glenda Mary Davison

**Affiliations:** ^1^ Department of Component Processing Western Cape Blood Service Cape Town South Africa; ^2^ Cape Peninsula University of Technology Cape Town South Africa; ^3^ SAMRC/CPUT Cardiometabolic Health Research Unit, Cape Peninsula University of Technology Cape Town South Africa

**Keywords:** chronic blood transfusion, haemolysis, neocytes, young red blood cells

## Abstract

**Background and Objectives:**

Red blood cell (RBC) concentrates are stored at 1–6°C for up to 42 days, but storage lesions can lead to wastage. Pooled neocytes may extend RBC shelf‐life, benefiting patients who require frequent transfusions. This study aimed to improve the longevity of stored RBCs by isolating neocytes and comparing the rate of haemolysis, biochemical changes and viability with filtered blood.

**Materials and Methods:**

Thirty filtered units were processed. Neocytes were extracted and enriched with saline‐adenine‐ glucose‐mannitol. Both filtered and neocyte‐enriched units were stored for 42 days. Samples were analysed every 14 days for RBC count, mean cell volume (MCV), mean cell haemoglobin concentration (MCHC), haemoglobin, sodium and supernatant haemolysis.

**Results:**

There was no significant difference in red cell count between filtered and neocyte‐enriched units (*p* = 0.27). Both types showed increased mean corpuscular volume and decreased MCHC over the 42 days, with no significant differences observed (*p* ≥ 0.05). Sodium levels in the supernatant decreased while percentage supernatant haemolysis increased steadily in both units, albeit without significant differences (*p* ≥ 0.05). The haemoglobin remained stable for both unit types.

**Conclusion:**

Overall, neocyte‐enriched blood did not demonstrate any longevity advantage compared to pre‐stored leucocyte‐reduced RBCs using the conventional manual collection method. These findings align with previous studies using various neocyte collection methods. Feasibility was highlighted as the main challenge, as many of these methods have proven too expensive and laborious.

**Highlights**

- Chronic red blood cell (RBC) transfusions are essential for treating conditions such as sickle cell disease and β‐thalassaemia. Neocyte‐enriched blood may prolong the interval between transfusions.
- The results of this study have shown no significant difference in RBC storage lifespan between neocyte‐enriched blood and filtered blood.
- These results highlight the need to reassess the use of neocyte‐enriched blood in chronic transfusions and explore more cost‐effective collection methods.

## INTRODUCTION

Annually, nearly 8 million people require blood transfusion in sub‐Saharan Africa (SSA), resulting in most countries experiencing a blood supply shortage [1, 2, 3]. Despite technological advancements, this shortage remains a significant challenge and requires a multi‐pronged approach, with several solutions being considered in managing blood stock [2, 4].

Prolonged storage of red blood cells (RBCs) may result in haemolysis post transfusion. This process leads to the release of haemoglobin and iron, which can damage blood vessels and mediate pathogen proliferation, thus increasing the risk of death [5]. To reduce this risk, the European Union (EU) has set the haemolysis limit to 0.8% [6]. During red cell haemolysis, potassium and free haemoglobin are released into the circulation, which can cause significant complications post transfusion. Potassium is vital in the heart's normal sinus rhythm, while elevated free haemoglobin indicates increased haemolysis [7]. For blood transfusion services to provide the best clinical outcome and reduce the adverse effects of transfusion, it is essential that the quality and stored life span of red cell products are constantly enhanced.

Patients with chronic anaemias, such as thalassaemia, sickle cell disease (SCD) or Diamond–Blackfan anaemia (DBA), often require regular blood transfusions [8, 9, 10]. However, repeated transfusions can lead to iron overload (haemochromatosis), which, if not medically managed, results in iron accumulation in the tissue and organ failure (haemosiderosis) [11]. Furthermore, these patients risk becoming alloimmunized and developing antibodies against antigens such as Kell and Duffy. These immune reactions can lead to cardiac failure, kidney failure and an increased risk of mortality [12]. The risk of infectious disease transmission, including human immunodeficiency virus (HIV), hepatitis B virus (HBV), hepatitis C virus (HCV) and syphilis, is also increased due to frequent donor exposure [13].

A promising approach to improving transfusion efficacy is the use of young RBCs or neocytes that have a longer lifespan. Initial studies using neocyte‐enriched blood showed encouraging results [14, 15, 16, 17, 18], while a recent in vivo study reported a mean interval increase from 26.1 to 45.8 days among thalassaemic patients [19]. In both research studies, 12 patients participated, and the conventional method of pooling neocytes was used.

Despite the prevalence of haemoglobinopathies and the need for chronic blood transfusion in Africa, limited research has focused on improving RBC viability. This study aimed to isolate young red cells or neocytes and examine their in vitro rate of haemolysis, haemoglobin, red cell count, red cell parameters, biochemical changes and viability as measures of longevity. The results were compared with those using the conventional filtered blood (leucocyte‐poor) pre‐stored over the standard 35–42‐day period.

## MATERIALS AND METHODS

### Study design

This prospective cohort study compared conventional filtered blood and neocyte‐enriched blood from 30 group‐AB donors at the headquarters of the Western Cape Blood Service (WCBS) in Ndabeni, Cape Town. Blood donations followed standard procedures, with a minimum interval of 56 days. All donated blood samples that screened positive for HIV, HBV, HCV or syphilis were excluded. The study focused on AB‐positive units owing to their low demand. After separating the neocyte red cell population from the filtered blood unit, multiple parameters were tested at 14‐day intervals to determine the level of haemolysis and longevity of the RBCs. These results were compared with those of the original filtered blood stored under the same conditions.

### Blood donation

A plasma low‐haemoglobin (Hb) analyser (Hemocue, Sweden) was used to determine the haemoglobin level before donation. A donor was allowed to donate if the haemoglobin level was above the required level of 12.5 g/dL. Blood was collected into a donation pack (Terumo, USA), two 10‐mL tubes and one 6‐mL ethylenediaminetetraacetic acid (EDTA) tube (Beckton Dickinson, USA). The 10‐mL tubes were used for viral testing, while the 6‐mL tube was used for RBC serology, haematology and syphilis testing. The donation process continued until the required whole blood (WB) volume of 450 mL was reached.

### Processing of WB

WB was collected from 30 regular, healthy donors who provided informed consent. Blood was collected into three satellite bags in Terumo quadruple blood bags. All units were weighed before processing and were processed within 24 h. The WB collected met the specifications required for component processing, with the final red blood cell concentrate (RBCC) volumes being within 250–350 mL (pre‐filtration) and 210–310 mL (post‐filtration). WB was stored at 20–24°C immediately after collection. The temperature was reduced to 2–6°C during WB processing. Centrifugation was performed using a Sorvall RC 12BP9+ centrifuge (Thermo Fisher Scientific, USA) at 4500 relative centrifugal force (RCF) for 12 min, maintaining a temperature of +4°C. The units were then separated into plasma, platelets and RBCC using the Terumo T‐ACE extractor. The RBCC pack contained 100 mL of the additive solution, saline‐adenine‐glucose‐mannitol (SAGM).

### Filtration of units

Once the RBCC unit was collected, the required transfer bags, sample packs and sample tubes were labelled appropriately. Each unit required 5 × 4‐mL EDTA samples (one for reticulocyte count and four for full blood cell count), 4 × 10‐mL EDTA (separation of plasma from RBCs) and 8 × 5‐mL cryo tubes (four for plasma sodium testing and four for supernatant haemolysis testing). Each unit also required eight sample packs (four for neocyte‐enriched blood and four for filtered blood), two transfer bags (one for neocyte‐enriched blood and one for SAGM) and an Imugard III‐RC filter pack (Terumo). After collection, the unit was sealed with an Imugard III‐RC filter pack using a Terumo sterile tubing welder (TSCD‐II). After approximately 10 min, the unit filtration was complete, and the filtered product was labelled as filtered blood. Approximately 50 mL of filtered blood was transferred into a separate transfer bag and used as the control for the study.

### Isolation of neocytes

**FIGURE 1:**
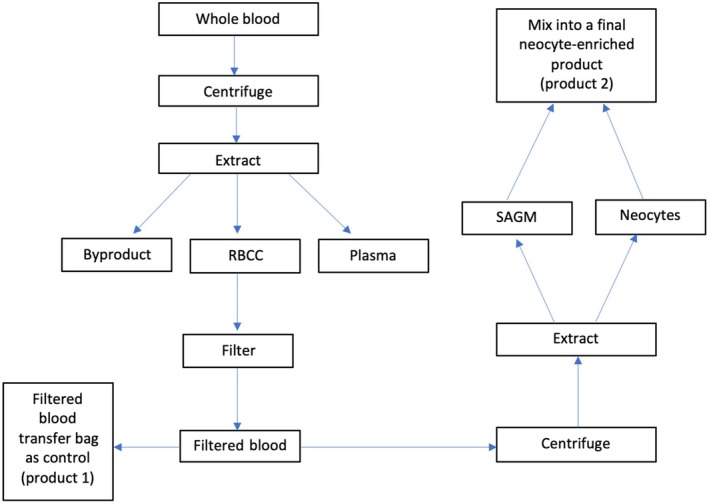
Flow diagram of methodology to extract neocytes. RBCC, red blood cell concentrate; SAGM, saline‐adenine‐glucose‐mannitol.

To isolate and enrich the neocyte population, two transfer bags were attached to the filtered blood bag using the TSCD‐II. These transfer bags were used to extract the neocytes and the SAGM from the filtered blood. The filtered blood unit with the attached bags was spun at 4500 RCF for 12 min, maintaining a temperature of +4°C in the Sorvall RC 12BP9+ centrifuge. The blood was separated using density gradient centrifugation. In this technique, particles, cells and components move through the gradient until they reach a point matching the medium's density. After centrifugation, the two transfer bags were placed on separate scales, and the scales were tared. The tube leading to the neocyte transfer bag was sealed using a manual clamp. A manual extractor was used to remove SAGM from the filtered blood bag into the SAGM transfer bag. The SAGM transfer tube was manually clamped. The tube leading to the SAGM transfer bag was sealed using the Terumo T‐Seal‐II, and the manual clamp leading to the neocyte transfer bag was removed. Approximately 35 g of the top 30% of the remaining RBCs was extracted into the neocyte transfer bag. The neocyte transfer tube was manually clamped and sealed using the Terumo T‐Seal II. The sealed tubes of both neocyte and SAGM transfer bags were welded together using the TSCD‐II. A fixed ratio (1:2.6) of SAGM to neocytes was applied, after approximately 13.46 g of SAGM was transferred from the SAGM transfer bag into the neocyte transfer bag. Once the SAGM was added, the transfer tube between the two bags was sealed using the Terumo T‐Seal‐II. The final volume of neocyte‐enriched blood was approximately 48.46 g. The SAGM transfer bag was discarded, and the neocyte bag was the final product. This means that both filtered blood and neocyte‐enriched blood were isolated from the same WB unit. This process is shown in Figure 1. Thereafter, the neocytes and the conventional filtered blood were stored for 42 days and tested every 14 days.

### Laboratory analysis

A serum separator tube (SST) was filled with 2 mL of blood, and sodium levels were analysed using the Architect c8000 analyser (Abbott, USA), while blood collected into an EDTA tube was used for haematological tests. The full blood count was performed using the Sysmex, XN‐3000 and the Sysmex, XN‐1000 (Sysmex, Kobe, Japan) systems. The Sysmex XN‐1000 has an RBC/PLT channel, which reads the RBC and platelets. The last 2 mL of the sample bag was used for the free haemoglobin test, on days 1, 14, 28 and 42. Each time the samples were collected, they were centrifuged at 4000 rpm for 15 min. A small drop of the supernatant was pipetted onto a slide, filling the plasma/low‐Hb cuvette. The percentage supernatant haemolysis was calculated using the formula $\frac{\left(100-\mathrm{HCT}\right)\times \text{plasma haemoglobin}\;\left(g\;{\mathrm{dL}}^{-1}\right)}{\text{total haemoglobin}\;\left(g\;{\mathrm{dL}}^{-1}\right)}$.

### Statistical analysis

The results were transcribed into Excel version 16.84, and statistical analysis was performed with SPSS (version 28) for analysis of variance (ANOVA). Microsoft Visual Studio Code (version 1.60) was used for descriptive statistics and Spearman correlation analysis. Normality was assessed using the Shapiro–Wilk test.

Python, Pandas, Seaborn and Matplotlib were used for data analysis. The results from days 1, 14, 28 and 42 were analysed and compared between the neocyte‐enriched blood and filtered blood using the *t*‐test. The Spearman correlation coefficient measured variable correlations, while the ANOVA determined the overall statistical significance. A *p*‐value of <0.05 indicated significance, and a positive or a negative correlation was determined by *x*‐values above or below 0, respectively.

### Ethical clearance

All donors gave consent, and ethical approval was obtained from the Cape Peninsula University of Technology Health Research Committee (HREC) on 24 February 2021 (reference no: CPUT/HW‐REC 2021/H5).

## RESULTS

During this study, 30 neocyte‐enriched blood units and 30 filtered blood units from the same donor were processed and compared. Significant changes in the MCV, supernatant haemolysis and sodium levels were observed in both unit types over the 42‐day storage period. A significant increase in the MCV and percentage supernatant haemolysis was observed. Sodium levels were highly variable but steadily decreased in both groups. All other parameters remained stable. The Shapiro–Wilk test confirmed that the collected data exhibited a normal distribution. One‐way ANOVA showed no significant differences between means across time points. Variations within the results have been noted for MCV, MCHC, sodium and percentage supernatant haemolysis. These results are shown in Table 1.

**TABLE 1:** Mean and standard deviation results of filtered blood and neocyte‐enriched blood.

Analyte	Filtered blood				Neocyte‐enriched blood				*p*‐value	*F‐*ratio
	D1	D14	D28	D42	D1	D14	D28	D42		
	Mean (±SD)	Mean (±SD)	Mean (±SD)	Mean (±SD)	Mean (±SD)	Mean (±SD)	Mean (±SD)	Mean (±SD)		
Red blood cell count (10^6^/μL)	6.59 (0.53)	6.64 (0.57)	6.52 (0.53)	6.54 (0.60)	6.50 (1.00)	6.63 (1.03)	6.54 (1.09)	6.54 (1.03)	0.27	0.02
MCV (fL)	92.27 (6.39)	95.85 (6.47)	99.09 (6.83)	101.6 (6.48)	94.86 (6.46)	98.44 (6.49)	101.8 (6.70)	104.11 (6.47)	0.87	253.87
MCHC (g/dL)	30.29 (1.39)	29.11 (1.00)	28.3 (0.89)	27.53 (1.09)	29.32 (1.17)	28.33 (0.98)	27.43 (0.99)	26.9 (1.03)	0.44	3.89
Hb (g/dL)	18.29 (1.27)	18.46 (1.45)	18.23 (1.32)	18.28 (1.30)	18.03 (2.50)	18.41 (2.65)	18.15 (2.73)	18.20 (2.55)	0.75	0.05
Sodium (mmol/L)	141.1 (3.18)	124.37 (4.05)	114.07 (5.12)	107.13 (4.31)	140.13 (5.46)	121.57 (8.48)	110.87 (9.07)	105.90 (7.28)	0.14	0.65
Supernatant haemolysis (%)	0.15 (0.07)	0.28 (0.09)	0.44 (0.12)	0.64 (0.13)	0.19 (0.08)	0.33 (0.07)	0.51 (0.14)	0.72 (0.18)	0.65	7.52
Average reticulocyte (10^9^/L)					75.79					

Abbreviations: Hb, haemoglobin; MCHC, mean cell haemoglobin concentration; MCV, mean cell volume; SD, standard deviation.

Both neocyte‐enriched blood and filtered blood showed an increase in the mean RBC count between days 1 and 14. However, despite a further decline in RBC by day 42, no significant difference was observed (all *p* > 0.05). As expected, the reticulocyte count was 76 × 10^9^/L in the neocyte‐enriched blood, confirming the presence of young, immature red cells, and a steady increase in mean cell volume (MCV) was observed in both the filtered blood and neocyte‐enriched blood. Although the MCV of the neocyte‐enriched blood was consistently higher, the values at each time point were not significantly different from that of the filtered blood (*p* > 0.05). However, a high variation in the results was observed (*F*‐ratio = 253.87). MCHC steadily decreased in both blood types over the 42‐day study period, reflecting the change in red cell morphology. Although the MCHC of both the neocyte‐enriched and filtered RBC was within the reference range, levels were significantly lower in the neocyte‐enriched blood at day 1 (*p* = 0.004), day 14 (*p* = 0.003), day 28 (*p* = 0.001) and day 42 (*p* = 0.02). Significantly high variability was observed for the MCHC (*F*‐ratio = 3.89). Haemoglobin levels in the neocyte‐enriched blood and filtered blood fluctuated significantly at each time point. Nevertheless, the two blood types had no significant difference in the haemoglobin levels.

Sodium levels decreased steadily in both types; however, the filtered blood had consistently higher levels at each time point, but this was not significant. The percentage change decreased between days 1 and 42 for both filtered blood and neocyte‐enriched blood. By day 42, filtered blood had an average sodium level of 107.93 mmol/L and neocyte‐enriched blood had 105.90 mmol/L. No significant difference was found between neocyte‐enriched blood and filtered blood for the overall sodium levels (*p* = 0.14); nor could any significant difference be identified at the individual time points between the two types. Nevertheless, considerable variation was observed (*F*‐ratio = 0.65).

Supernatant haemolysis increased steadily throughout the 42 days. The neocyte‐enriched blood had a higher percentage of supernatant haemolysis than filtered blood at each time point. By day 42, the filtered blood had 0.64%, whereas the neocyte‐enriched blood had 0.72%. This represented a percentage change between days 1 and 42 of 0.49% for filtered blood and of 0.53% for neocyte‐enriched blood. However, overall, no significant difference was observed between the neocyte‐enriched and filtered blood (*p* = 0.65). When examining each time point, significant differences were seen. These were at day 1 (0.02), day 14 (0.02) and day 28 (0.04). Significantly high variances were observed in all the results (*F* = 7.52).

### Correlation studies

**FIGURE 2:**
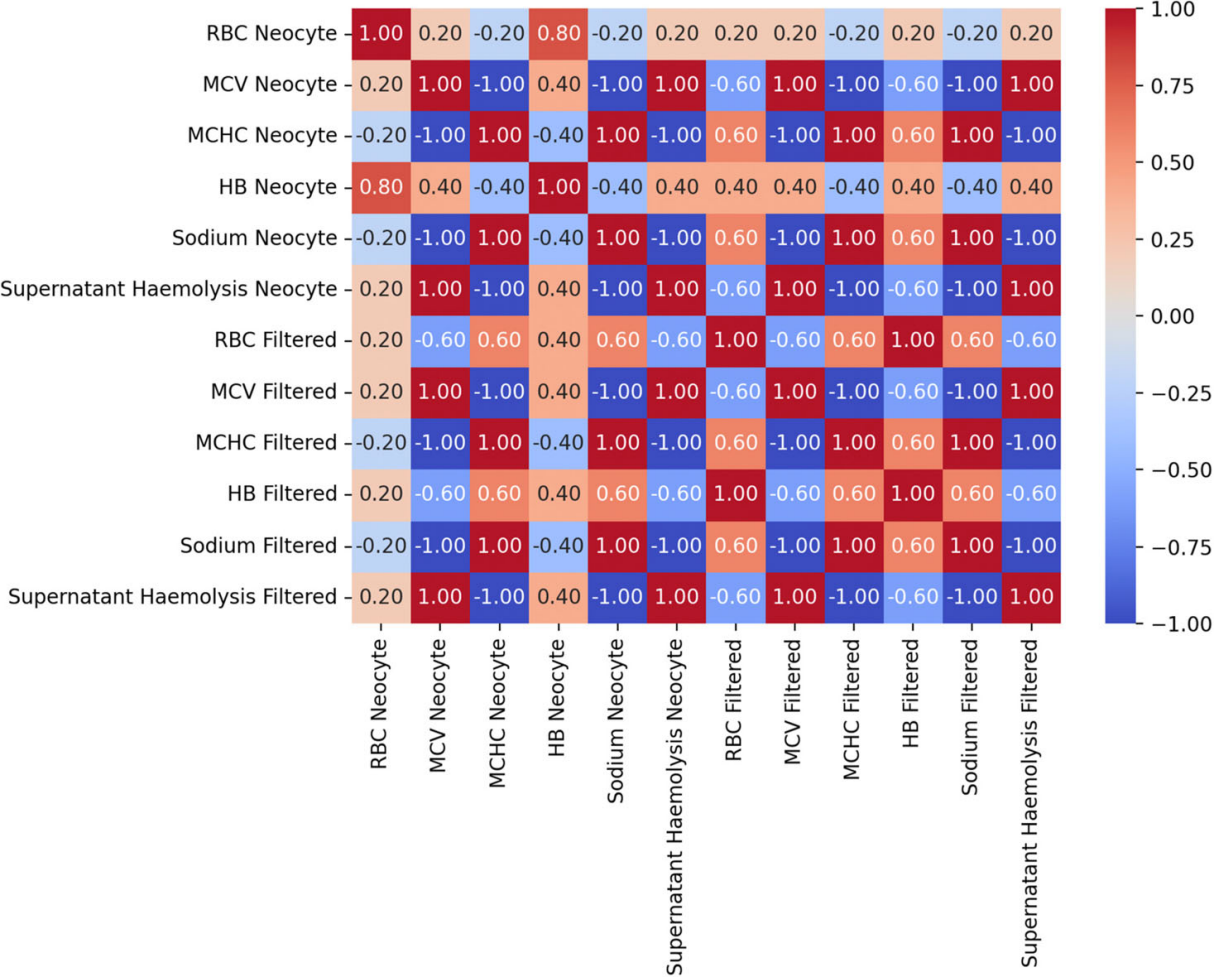
Heatmap of the Spearman correlation coefficient between neocyte‐enriched blood and filtered blood. Hb, haemoglobin; MCHC, mean cell haemoglobin concentration; MCV, mean cell volume; RBC, red blood cell.

A Spearman correlation was also performed between all variables. Results that are above 0 represent a positive correlation, while results below 0 represent a negative correlation. The heatmap is presented in Figure 2.

As expected, significant positive correlations were observed between the MCV, MCHC, sodium levels and supernatant haemolysis (*x* = 1.00) in both neocyte‐enriched blood samples and leucocyte‐poor blood samples, suggesting a predictable relationship. Haemoglobin levels had a weaker correlation (*x* = 0.60), which was still significant.

## DISCUSSION

In this study of 30 group‐AB units of blood processed as conventional filtered blood and neocyte‐enriched blood, the objective was to investigate whether enriching for neocytes would prolong the in vitro longevity of RBCs, which may result in less frequent transfusions. Our results indicate that despite variations at different time points, there is no significant difference in the longevity of the neocytes as measured by haemolysis, sodium levels and red cell parameters.

In South Africa, the standard storage time for RBCs is 42 days, and because of the red cell ageing process, approximately 25% of the cells are removed by the spleen after transfusion [20]. RBCs undergo significant changes during storage, which include losing their biconcave shape, leading to an increase in echinocytes and abnormally shaped RBCs [21]. Multiple biochemical changes also occur, including intracellular potassium loss and increased sodium. Consequently, the red cell volume increases, leading to changes in the MCV and MCHC. RBCs rely on anaerobic glycolysis to generate energy, which produces adenosine triphosphate (ATP), 2,3‐diphosphoglycerate (2,3‐DPG) and reduced nicotinamide adenine dinucleotide (NADH) 2,3‐DPG. This process slows significantly during storage, causing the red cells to lose the ability to release oxygen [22, 23, 24, 25]. Prolonged oxidative stress leads to irreversible damage, such as lipid and protein breakdown, and ultimately removes the RBCs from the circulation [26].

Multiple studies have indicated that extended RBC storage before transfusion is associated with an increase in mortality, a higher risk for infections, as well as multiple organ failure [27, 28]. Furthermore, using aged RBCs and more frequent transfusions increase the risk of iron overload, reduced oxygen transportation and pH levels and increased breakdown of RBCs [29]. Therefore, by transfusing younger RBCs, many risks could be prevented and iron overload could be limited [29]. Multiple parameters were assessed in this study to determine whether neocyte‐enriched blood, collected using the conventional top and bottom system, improved the longevity of RBCs. However, no significant differences or improvements were found.

Several research studies have used various methods for collecting young RBCs and assessed the red cell senescence through different parameters [16, 30, 31, 32]. Some of these methods include the conventional top‐and‐bottom method and the apheresis method. The conventional method involves centrifuging blood to separate the different RBC populations based on density. This method is cost effective and simple and requires minimal specialized equipment. However, it offers limited precision in isolating the young RBCs and can cause potential mechanical damage due to multiple centrifugation steps. In contrast, apheresis is an automated process that selectively collects RBCs based on their size and density. This process is much more precise in collecting the desired RBCs and reduces the likelihood of causing mechanical cellular damage.

Nevertheless, apheresis is expensive and can be less feasible. Therefore, while the conventional method is simple, more accessible and cost effective, apheresis provides greater accuracy and cell integrity, but at a higher cost and operational complexity. Previous studies in France, Italy and India have pooled neocyte‐enriched blood from multiple units [18, 19]. In contrast, the neocyte component prepared in this study was not pooled, which may limit its direct comparison with standard components. However, this study investigated the in vitro longevity of the neocyte population based on cellular and functional analysis.

Multiple research studies have been conducted to investigate the storage of young RBCs. As expected, contrasting results have been found. For instance, a study in Italy assessed the viability of young, middle‐aged and older RBCs. Blood samples were collected in K‐EDTA vacutainer tubes and centrifuged to separate the different blood populations. The results suggested that young RBCs are more sensitive to survival signals, such as erythropoietin, compared to older RBCs [31]. In contrast, with results similar to this current study, experiments conducted in Australia found no notable differences between young and old RBCs aged over 42 days. Filtered blood was centrifuged to separate the young and old RBCs [32]. However, the in vivo longevity of neocytes collected via apheresis has shown promising results, with an increase in red cell longevity [19]. These differences highlight the need for standardization in young RBC collection methods as well as monitoring of red cell senescence.

The results of this study have provided valuable insights into red cell senescence differences between neocyte‐enriched blood and filtered blood. The conventional top‐and‐bottom system for neocyte collection offers a more practical and cost effective choice than alternative methods such as the apheresis method. This study did have limitations, which included a small sample size. The study focused solely on the in vitro RBC storage and did not investigate in vivo survival. Moreover, only the top‐and‐bottom system was examined, and a comparison to the apheresis method was not conducted.

The use of flow cytometry has become an international standard for the identification of cell populations [33]. Some studies have suggested that the expression of CD47 decreases as red cells age and that young RBCs express CD35, CD44 and CD71 [34, 35]. In this study, reticulocyte count was used to determine the successful collection of neocytes; however, flow cytometry is recommended for more precise measurement. Despite these limitations, this study was able to demonstrate that using the conventional method to collect neocytes provides no significant survival advantages over the 42‐day storage period.

Research has revealed promising methods to improve and extend RBC storage beyond that achieved using conventional techniques. Novel additive solutions such as PAG3M, E‐Sol 5 and AS‐7 have higher levels of ATP and better maintenance of 2,3‐DPG levels during storage [36]. Furthermore, new cryopreservation methods have emerged, which include core–shell microfiber encapsulation, leading to higher RBC recovery without glycerol [37].

Future research should involve conducting studies with larger sample sizes to improve the statistical analysis and investigating the in vivo survival and clinical efficacy of neocyte‐enriched blood compared to filtered blood. Future studies should also explore alternative methods for neocyte collection to identify more efficient, cost‐effective and practical approaches. Finally, performing a cost–benefit and risk analysis in clinical practice will be crucial for understanding the short‐term and long‐term healthcare costs and risks.

## CONFLICT OF INTEREST STATEMENT

The authors declare no conflicts of interest.

## Data Availability

The data that support the findings of this study are available from the corresponding author upon reasonable request.
